# Detection of *Plasmodium knowlesi*, *Plasmodium falciparum* and *Plasmodium vivax* using loop-mediated isothermal amplification (LAMP) in a co-endemic area in Malaysia

**DOI:** 10.1186/s12936-016-1676-9

**Published:** 2017-01-13

**Authors:** Kim A. Piera, Ammar Aziz, Timothy William, David Bell, Iveth J. González, Bridget E. Barber, Nicholas M. Anstey, Matthew J. Grigg

**Affiliations:** 1 Global and Tropical Health Division, Menzies School of Health Research and Charles Darwin University, Darwin, NT Australia; 2 Infectious Diseases Society Sabah-Menzies School of Health Research Clinical Research Unit, Kota Kinabalu, Sabah Malaysia; 3 Jesselton Medical Centre, Kota Kinabalu, Sabah Malaysia; 4 Clinical Research Centre, Queen Elizabeth Hospital, Kota Kinabalu, Sabah Malaysia; 5 Global Good Fund/Intellectual Ventures Laboratory, Bellevue, WA USA; 6 FIND, Geneva, Switzerland

**Keywords:** LAMP, *Plasmodium knowlesi*, Malaria, Diagnosis, RDT, PCR

## Abstract

**Background:**

*Plasmodium knowlesi* is the most common cause of malaria in Malaysia. However, microscopic diagnosis is inaccurate and rapid diagnostic tests (RDTs) are insufficiently sensitive. PCR is sensitive and specific but not feasible at a district level. Loop-mediated isothermal amplification (LAMP) shows potential with only basic requirements. A commercially available LAMP assay, the Eiken Loopamp™ MALARIA Pan Detection kit, is sensitive for *Plasmodium falciparum* and *Plasmodium vivax*, but has not previously been evaluated for *P. knowlesi*. This study aims to determine the sensitivity of this LAMP assay for detecting *P. knowlesi* infection.

**Methods:**

Study participants included 73 uncomplicated malaria patients with PCR species confirmation: 50 *P. knowlesi*, 20 *P. falciparum* and 3 *P. vivax*. Nineteen malaria-negative, non-endemic area controls were also included. The sensitivity of the Eiken Loopamp™ MALARIA Pan Detection kit (Pan LAMP) for detecting each *Plasmodium* species was evaluated. Sensitivity and specificity of the Eiken Loopamp™ MALARIA *Pf* Detection kit (*Pf* LAMP) for *P. falciparum* were also determined. The limit of detection for each LAMP assay was evaluated, with results compared to PCR. All *P. knowlesi* patients were also tested by CareStart™ (Pf/VOM) and OptiMAL-IT™ (Pan/Pf) RDTs.

**Results:**

The sensitivity of the Pan LAMP assay was 100% for *P. knowlesi* (95% CI 92.9–100), *P. falciparum* (95% CI 83.2–100), and *P. vivax* (95% CI 29.2–100). The *Pf* LAMP was 100% sensitive and specific for *P. falciparum* detection, with all *P. knowlesi* samples having a negative reaction. LAMP sensitivity was superior to both RDTs, with only 10 and 28% of *P. knowlesi* samples testing positive to CareStart™ and OptiMAL-IT™, respectively. Limit of detection using the Pan LAMP for both *P. knowlesi* and *P. vivax* was 2 parasites/μL, comparable to PCR. For *P. falciparum* both the Pan LAMP and *Pf* LAMP demonstrated a limit of detection of 20 parasites/μL.

**Conclusions:**

The Eiken Loopamp™ MALARIA Pan Detection kit is sensitive for detection of *P. knowlesi* in low parasitaemia clinical infections, as well as *P. falciparum* and *P. vivax.* However, a *P. knowlesi*-specific field assay in a simpler format would assist correct species identification and initiation of optimal treatment for all malaria patients.

## Background

*Plasmodium knowlesi* has emerged as the most common cause of malaria infection in Malaysia [1–3]. It can cause severe and fatal disease and, due to its rapid clinical course, is important to diagnose and treat early [4–8]. Microscopy is the diagnostic method of choice in Malaysia but has limitations for *P. knowlesi* as ring stages resemble *Plasmodium falciparum* and later trophozoites resemble *Plasmodium malariae* [8–10], hence misidentification is common. *P. knowlesi* causes fever at low parasitaemia [4, 6] and, therefore, may be undetected by microscopy. Better methods for the diagnosis of knowlesi malaria are needed.

There have been no immunochromatographic rapid diagnostic tests (RDTs) specifically designed for *P. knowlesi* detection. Commercially available RDTs measuring an antibody response to non-specific pan malarial antigens, combined with the variable cross-reactive response of *P. knowlesi* to *Plasmodium vivax* or *P. falciparum*-designed antigen capture test components have been investigated previously [11–13]. However, the sensitivity of these RDTs for *P. knowlesi* detection is poor, particularly at low parasitaemia, and they are not currently suitable for clinical use.

Molecular methods, such as PCR, are sensitive and specific [14, 15], but are not feasible in many malaria-endemic areas due to a lack of resources, and do not allow rapid diagnosis for correct initiation of treatment. Loop-mediated isothermal amplification (LAMP) is a promising molecular diagnostic technique that involves nucleic acid amplification and target gene detection in a single step, and is applicable for use in district hospital laboratories as it is sensitive, specific and easy to use. LAMP does not require expensive reagents or equipment and only minimal training is needed [16, 17].

The Eiken Loopamp™ MALARIA Pan Detection kit (Pan LAMP) is a field-stable kit suitable for use in rural health facilities, designed to detect a non-specific *Plasmodium* genus target, and has been shown to be sensitive for both *P. falciparum* and *P. vivax*, including in clinical samples with low parasite counts [18–20]. However, it has not previously been validated for the detection of *P. knowlesi*. This study evaluated the sensitivity of the Pan LAMP kit in uncomplicated knowlesi malaria using clinical samples to determine whether it might be a useful diagnostic or surveillance tool. In addition, the limit of detection of this assay was determined for *P. knowlesi*, *P. falciparum* and *P. vivax* samples. Finally, the Eiken *P. falciparum*-specific LAMP (*Pf* LAMP) for diagnostic sensitivity and specificity of *P. falciparum* infection was also evaluated.

## Methods

### Study sites

This study was conducted with samples collected from three sites in Sabah, Malaysia: Kudat District Hospital, Kota Marudu District Hospital and Queen Elizabeth Hospital in Kota Kinabalu.

### Sample collection

Patients with *P. knowlesi* and *P. vivax* patients were prospectively enrolled as part of previously reported clinical drug trials in three district hospitals in Sabah, Malaysia [21, 22]. *P. falciparum* patients were enrolled as part of an ongoing multicentre surveillance study for in vivo artesunate resistance in Sabah.

There were 50 *P. knowlesi*, 20 *P. falciparum* and 3 *P. vivax* patients included in the study with PCR-confirmed mono-infections, all of whom were microscopy positive and enrolled prior to treatment. In addition, 19 healthy human volunteers from a non-endemic country (Australia) with no previous malaria exposure were included as controls after written informed consent. Severe malaria was defined according to WHO 2014 guidelines [23]. Heparinized whole blood was collected and frozen at −80 °C until analysis.

### LAMP

The Pan LAMP assay was performed on all clinical malaria samples and negative controls, and the *Pf* LAMP on all except the *P. vivax* samples. The LAMP assays were conducted strictly according to the Eiken Loopamp™ MALARIA Pan and *Pf* Detection kit procedures (Eiken Chemical Company, Japan). Briefly, DNA was extracted using a boil and spin method where 60 μL of thawed, well mixed whole blood was added to a labelled tube containing 60 μL of extraction buffer (400 nM NaCl, 40 mM Tris pH 6.5, 0.4% SDS), vortexed and incubated in a heat block at 95 °C for 5 min. The tubes were centrifuged at 10,000*g* for 3 min and 30 μL of supernatant was added to 345 μL sterile distilled water. After mixing well, 30 μL was added to the malaria Pan or *Pf* reaction tube with one negative and one positive control included in each 16 reactions, mixed well by inversion to reconstitute primers in the lid of the reaction tubes and incubated at 65 °C for 40 min in the LA-500 real time turbidimeter (Ref: MVL300—Eiken Chemical Co) before polymerase inactivation at 80 °C for 5 min.

A positive malaria result using the LAMP reaction can be visualized as a magnesium pyrophosphate precipitate detectable by validated turbidimeter. All reactions were also visualized manually by the operator for fluorescence and turbidity, with a subsequent reading by a separate person blinded to previous LAMP readings and PCR results.

### PCR

DNA was extracted from 200 μL of blood using Qiagen’s QIAamp DNA Blood mini kit™ as per the manufacturer’s recommendations. *Plasmodium* species and limit of detection samples were confirmed using a non-multiplexed version of the PCR assay previously described by Padley et al. with primers for *P. falciparum*, *P. vivax*, *P. malariae*, and *P. ovale* [24] and for *P. knowlesi* using the PCR method published by Imwong et al. [14]. All limit of detection samples were additionally tested by nested PCR according to the method published by Snounou et al. [25].

### RDTs

All *P. knowlesi* patients in this study had whole blood tested using both the OptiMAL-IT™ RDT (WHO RDT catalogue #710024) containing *Plasmodium* genus pan-pLDH and *P. falciparum*-specific pLDH test components, and the CareStart™ Malaria HRP2/pLDH (Pf/VOM) Combo RDT (#G0171) containing a *P. falciparum* HRP2 and non-*P. falciparum* pLDH combination as per manufacturer’s recommendations. RDTs were selected for potential combined specificity for *P. knowlesi* detection [12].

### Limit of detection

Samples with known parasite counts per microlitre of whole blood, determined by an expert research microscopist, were used to test the limit of detection of the Pan LAMP assay for *P. knowlesi, P. falciparum* and *P. vivax*, and the *Pf* LAMP assay for *P. falciparum*. Three samples of each relevant *Plasmodium* spp. were chosen. Frozen whole blood samples were thawed then diluted in fresh, malaria negative, non-endemic country whole blood to produce parasitaemias equivalent to 2000, 200, 20, 2, and 0.2 parasites/μL with the diluting blood serving as a negative control. These samples then had parasite DNA extracted using the different methods described above for each LAMP assay and conventional PCR, respectively.

### Statistical analysis

Data were analysed by using STATA, version 12 (StataCorp LP, College Station, TX, USA). Between-group differences were compared with Wilcoxon–Mann–Whitney test for non-parametric data. PCR results were used as the gold standard. Test sensitivity was defined as true positives/(true positives + false negatives), and test specificity was defined as true negatives/(true negatives + false positives); 95% confidence intervals were calculated using Wilson’s method.

## Results

### Baseline characteristics

Demographic, clinical and parasitological characteristics of the patients are shown in Table 1. Median (IQR) parasitaemia was 1197/μL (300–3920) and 7246/μL (2299–17,232) for those with *P. knowlesi* and *P. falciparum* infection, respectively (p < 0.001).

**Table 1:** Baseline characteristics, LAMP assay and RDT results

Variable	*P. knowlesi*	*P. falciparum*
No. participants	50	20
Age		
Median	32	36
(IQR) [range]	(19–49) [3–72]	(28.5–46.5) [19–73]
Gender		
Number male	38	13
%	76	65
Parasite count/μL		
Geometric mean	1197	7246
(IQR) [range]	(300–3920) [43–46350]	(2299–17232) [360–190912]
Severe		
n (%)	0 (0)	4 (20)
Pan LAMP		
Number positive (%)	50 (100)	20 (100)
Sensitivity % (95% CI)	100 (92.9–100)	100 (83.2–100)
Limit of detection (parasites/μL)	2.0	20.0
*Pf* LAMP		
Number positive (%)	0 (0)	20 (100)
Sensitivity % (95% CI)	0 (0)	100 (83.2–100)
Specificity % (95% CI)	0 (0)	100 (94.8–100)
Limit of detection (parasites/μL)	–	20.0
RDTs		
OptiMAL-IT® Pan pLDH		
n (%) [95% CI]	5 (10) [3.3–21.8]	–
OptiMAL-IT® *Pf* pLDH		
n (%) [95% CI]	3 (6) [1.3–16.5]	–
CareStart® non-*Pf* pLDH		
n (%) [95% CI]	14 (28) [16.2–42.5]	–

### LAMP

All 50 *P. knowlesi* samples were positive on the Pan LAMP and negative on the *Pf* LAMP. The 20 *P. falciparum* samples were positive in both assays. The 3 *P. vivax* samples were positive in the Pan LAMP assay. All 19 controls were negative in both assays. This gave a sensitivity of 100% for the Pan LAMP for *P. knowlesi* (95% CI 92.9–100), *P. falciparum* (95% CI 83.2–100) and *P. vivax* (95% CI 29.2–100). When combining results for *Plasmodium* genus detection overall, the Pan LAMP had both 100% sensitivity (95% CI 95.1–100) and specificity (95% CI 82.4–100). For *P. falciparum* detection, the *Pf* LAMP also demonstrated both 100% sensitivity (95% CI 83.2–100) and specificity (95% CI 94.8–100). All manual fluorescence and turbidity readings were 100% concordant between observers and with the LAMP turbidimeter results.

### Limit of detection

The limit of detection for *P. knowlesi* for both the Pan LAMP and specific PCR assays was 2 parasites/μL, with 6/12 and 2/4 (50%) of replicates for each method, respectively, also positive at 0.2 parasites/μL. For *P. falciparum,* the Pan LAMP limit of detection was 20 parasites/μL, with 8/12 (67%) and 5/12 (42%) of replicates also positive at 2 and 0.2 parasites/μL, respectively, compared to a limit of detection of 2 parasites/μL using specific PCR, with 3/4 (75%) of replicates also positive at 0.2 parasites/μL. For *P. vivax* the limit of detection for both methods was 2 parasites/μL, with 9/12 (75%) and 2/4 (50%) of replicates also positive at 0.2 parasites/μL for the Pan LAMP and specific PCR assays, respectively. For *P. falciparum* samples, the *Pf* LAMP also had a limit of detection of 20 parasites/μL, with 3/6 (50%) of replicates also positive at 2 parasites/μL.

### RDTs

For the *P. knowlesi* samples, the sensitivity of the pan-pLDH test component in the OptiMAL-IT™ RDT was 10% (95% CI 3.3–21.8; 5/50) and the CareStart™ non-*P. falciparum* pLDH was 28% (95% CI 16.2–42.5; 14/50). Three of the five *P. knowlesi* samples with a positive pan-pLDH in the OptiMAL-IT™ RDT also tested positive to the *P. falciparum*-specific pLDH with the same band intensity.

## Discussion

The Eiken Loopamp™ MALARIA Pan detection kit is highly sensitive, although inherently non-specific, for the diagnosis of uncomplicated knowlesi malaria, with 100% sensitivity in the population studied, including clinical samples with parasite counts as low as 43 parasites/μL. The limit of detection for *P. knowlesi* using the Pan LAMP assay was low at 2 parasites/μL, comparable to PCR, and well below the threshold of detection for standard microscopy of approximately 50 parasites/μL [26]. Similar limits of detection were found for *P. falciparum* and *P. vivax*, which were also comparable to PCR. Sensitivity of the Pan LAMP for *P. knowlesi* detection far exceeded that of pLDH-based RDTs (OptiMAL-IT™ and CareStart™), with the sensitivity of these RDTs too low for clinical use, as previously reported [12].

As described with other LAMP systems, the technical benefits of this LAMP assay include its high sensitivity, stability at ambient temperature, and relatively simple use compared to other molecular diagnostic methods [16, 17]. It requires no specialized equipment and minimal training, and is suitable for use in basic health facilities where accurate *Plasmodium* species confirmation would help to guide public health reporting, disease surveillance and in countries such as Malaysia, monitoring of national malaria elimination goals by 2020 [27].

This assay does however have some limitations. By definition, it is non-specific, with Pan LAMP shown to detect *P. falciparum* [18] and *P. vivax* [19], and therefore does not allow differentiation of *P. vivax*-infected patients requiring additional liver-stage treatment. Nor does it identify co-infection in co-endemic areas [1, 28], hence the Pan LAMP does not offer an improvement in diagnostic specificity. The study design would also be improved by conducting this evaluation in a field setting with fresh blood samples, however an expected decrease in sensitivity for either assay using thawed blood samples was not evident.

In the absence of appropriately sensitive and specific monoclonal antibodies for RDT, a specific *P. knowlesi* LAMP would aid diagnosis and surveillance in regional areas. Currently three LAMP assays have been designed specifically for *P. knowlesi*, targeting the β-tubulin [29], AMA-1 [30] and mitochondrial genes [17], respectively, with high sensitivity and specificity reported. However, these assays have not yet been utilized for clinical or surveillance purposes in endemic countries, particularly where PCR is available.

## Conclusion

The Eiken Loopamp™ MALARIA Pan Detection kit had excellent sensitivity for the diagnosis of uncomplicated *P. knowlesi* infection, even in low parasitaemia samples, and may be a suitable tool for malaria surveillance. The limitations of current RDTs and microscopy for *P. knowlesi* diagnosis means the development of a specific, field-applicable, inexpensive rapid diagnostic tool to improve clinical malaria diagnosis in *P. knowlesi*-endemic countries, such as Malaysia, remains an important challenge.
